# Letter on the Insertion and Preparation of Natural Teeth

**Published:** 1852-01

**Authors:** 


### LETTER ON THE PREPARATION AND INSERTION OF NATURAL TEETH.

THE following extract of a letter from a friend, we give, hoping it may elicit such information as is desired. Whether the human teeth could be much improved in the manner suggested or not, we cannot say. We know that such experiments have been made on wood, so as to preserve it from decay; and, if we mistake not, similar experiments have been made on the hippopotamus' tusk, so that it should resist the action of the secretions of the mouth, but we cannot now refer to the name of the individual, or the particular process to accomplish this.

We occasionally insert the bicuspids on pivot, and always use the natural teeth for this purpose, and have a few patients who prefer them for the incisors and canine. But for plate teeth, they cannot be made to answer so good a purpose. We sometimes find it impossible to get porcelain teeth to correspond with decayed and plugged natural teeth; and yet if we select good, sound natural ones, the same difficulty occurs.

DR. TAYLOR—DEAR SIR: One of *my* great troubles is, the selecting of *teeth* for insertion to match either plate or pivot, except when I *take* a *human* tooth, (which, by the by, is not very often,) and *then* I have no *trouble*, for they are yellow at the neck, and a transparent blue at the apex. What do you think of the *idea* of taking the *crowns* of *human teeth*, prepare them as they do porcelain teeth for market, by grinding the neck, drilling the pivot hole, &c., *and then* placing them under a powerful exhausting pump, and after exerting the full extent of its power let in *silisic acid*, and I think, in the course of a few hours or days, they will resist all the humors of the mouth, &c. I have no doubt the acid can be made under pressure, without the intervention of potassa. Your views, if convenient.

When you are required to insert a bicuspid with pivot, what kind of a *tooth* do *you use?* I, not being able to find porcelain to match, insert nothing but human teeth; and they are not always to be had, and, at present, too much trouble to prepare. Most respectfully, your obedient servant, I. F.

